# Novel *AQP2* Mutations and Clinical Characteristics in Seven Chinese Families With Congenital Nephrogenic Diabetes Insipidus

**DOI:** 10.3389/fendo.2021.686818

**Published:** 2021-06-10

**Authors:** Qian Li, Dan Tian, Jing Cen, Lian Duan, Weibo Xia

**Affiliations:** ^1^ Department of Endocrinology, Key Laboratory of Endocrinology, NHC, State Key Laboratory of Complex Severe and Rare Diseases, Peking Union Medical College Hospital, Chinese Academy of Medical Sciences, Beijing, China; ^2^ Department of Nuclear Medicine, The First Affiliated Hospital, College of Medicine, Zhejiang University, Hangzhou, China; ^3^ Department of Medical Cell Biology, Uppsala University, Uppsala, Sweden

**Keywords:** Nephrogenic diabetes insipidus, aquaporin-2, mutation, water resorption, vasopressin V2 receptor

## Abstract

**Objective:**

Mutations in *AQP2 (aquaporin-2)* lead to rare congenital nephrogenic diabetes insipidus (NDI), which has been limitedly studied in Chinese population.

**Methods:**

Twenty-five subjects from seven families with NDI in a department (Beijing, PUMCH) were screened for *AQP2* mutations. Clinical characteristics were described and genotype-phenotype correlation analysis was performed.

**Results:**

We identified 9 *AQP2* mutations in 13 patients with NDI, including 3 novel *AQP2* mutations (p.G165D, p.Q255RfsTer72 and IVS3-3delC). Missense mutations were the most common mutation type, followed by splicing mutations, and frameshift mutations caused by small deletion or insertion. The onset-age in our patients was younger than 1 year old. Common manifestations included polydipsia, polyuria (7/7) and intermittent fever (6/7). Less common presentations included short stature (3/7) and mental impairment (1/7). High osmotic hypernatremia and low osmotic urine were the main biochemical features. Dilation of the urinary tract was a common complication of NDI (3/6). Level of serum sodium in NDI patients with compound het AQP2 mutations was higher than non-compound het mutations.

**Conclusion:**

In the first and largest case series of NDI caused by *AQP2* mutation in Chinese population, we identified 9 *AQP2* mutations, including 3 novel mutations. Phenotype was found to correlate with genotypes, revealed by higher level of serum sodium in patients with compound het *AQP2* mutations than non-compound het mutations. This knowledge broadens genotypic and phenotypic spectrum for rare congenital NDI and provided basis for studying molecular biology of AQP2.

## Introduction

Congenital Nephrogenic Diabetes Insipidus (NDI) is characterized by impaired water resorption in the collecting duct due to insensitivity to arginine vasopressin (AVP) of principal cells, leading to polyuria and polydipsia (1). Mutations in the two key genes-*AQP2* (*aquaporin-2*) and *AVPR2* (*vasopressin V2 receptor*) were identified in most patients with a clear phenotype of NDI (2). Approximately 10% of patients with inherited NDI have a mutation in *AQP2* with an autosomal recessive or dominant pattern (OMIM 125800, 107777)) (3). The manifestations generally emerge within the first weeks of life. Infants with congenital NDI may suffer from hypernatremic dehydration, intermittent fever, irritaility, poor feeding and constipation (4). Repeated episodes of hypernatraemic dehydration and rehydration may cause brain tissue damage, seizure and even impair mental development (5, 6). Dilation of the urinary tract was present in 67% of the reported cases, which should be treated timely to avoid end-stage renal disease (7). Current therapeutic methods for NDI focus on relieving symptoms, which are limited and only partially beneficial (8).

AQP2 is a key factor for maintaining normal body water homeostasis. When the plasma osmolality increases, antidiuretic hormone (AVP) is released from the pituitary gland and binds to AVPR2 in the principal cells of the kidney collecting duct, resulting in accumulation of AQP2 in the apical plasma membrane, which is responsible for water reabsorption (9, 10). AQP2 forms a homotetramer and each monomer is composed of 271 amino acids, containing six transmembrane spanning regions with intracellular COOH terminus, which is essential for correct routing of AQP2. The structure of AQP2 has been clarified at 4.9-Å resolution, except for the structure of intracellular domains (11). The trafficking and degradation of AQP2 are regulated by multiple post-translational modifications, including phosphorylation, ubiquitination and glycosylation (12, 13). For example, AVP increases phosphorylation of AQP2 at ser256 and ser269, which is important for the accumulation of AQP2 (14–16). More than 90% of mutations are inherited in autosomal recessive mode, which can be categorized into three types according to the structural analysis: (i) the pore features (e.g. A70D), (ii) the tetramer assembly (e.g. T126M), and (iii) the monomer folding (e.g. A47V). The remaining 10% of mutations are dominant, mainly involving the C-terminal tail for AQP2 routing (e.g. R254L) (17–21).

In this study, we performed gene mutation analysis in 7 Chinese families and identified 9 *AQP2* mutations, including 3 novel *AQP2* mutations (p.G165D, p.Q255RfsTer72 and IVS3-3delC). Further, we characterized the clinical, biochemical and imaging features in these patients, and performed genotype-phenotype correlation analysis.

## Materials and Methods

### Subjects

All patients included in this study were referred to our department in Peking Union Medical College Hospital (PUMCH) for diagnosis and treatment of NDI. Diagnosis of NDI was made according to family histories, clinical symptoms (polyuria and polydipsia, fever due to dehydration), laboratory data (urine and serum osmolality, serum sodium), and responses to water deprivation and/or minirin administration. Patients were enrolled after approval from the Institutional Review Board and Ethics Committee of PUMCH and written informed consents were achieved.

### Clinical, Biochemical and Imaging Analyses

Medical records were reviewed to extract information, consisting of demographic data, manifestations, biochemical and imaging results. Short stature was defined as a condition in which the height of an individual is within the 3rd percentile of the height for Chinese population of the same age and sex (22). Mental impairment was assessed by qualified doctors. Estimated urine volume (L) in 24 hours was recorded according to detailed medical histories. Routine laboratory tests, including serum sodium (Na), creatinine (Cr(E)), uric acid (UA), serum and urine osmolality, and urine specific gravity (SG), were measured by auto-analyzers according to standard methods at the central laboratory of PUMCH. Reference ranges were provided by the central laboratory of PUMCH. Urological ultrasonography was performed in 6 of 7 patients by qualified doctors.

### Genomic DNA Extraction, Amplification, and Sequencing

Whole blood samples of patients and their family members were collected. Genomic DNA was purified using DNA extraction kit (QIAamp DNA Micro; Qiagen, Germany) according to the manufacturer’s protocol. Primers were designed targeting exons and flanking intron sequences of *AQP2* and *AVPR2*. The primer sets and annealing temperature are given in detail in **Supplementary Table 1**. All polymerase chain reactions (PCR) were performed using 100 ng of genomic DNA template in a total volume of 50 μl containing 10 pmol of each primer, and 25 μl of 2x Easytaq mix (Tiangen Biotech, Beijing, China), under the following conditions: initial denaturation at 94°C for 5 min, followed by 30 cycles at 94°C for 30 s, 60–63°C for 30 s and 72°C for 60 s, finally elongation at 72°C for 7 min. Direct DNA sequence analysis was performed by automated DNA sequencing in an ABI DNA sequencer (Model 377, Applied Biosystems, Foster City, CA), and data were compared with published sequences (AQP2: NC_000012, NM_000486, NP_000477; AVPR2: NC_000023, NM_000054, NP_000045).Gene SNPs (single nucleotide polymorphism) were excluded. In silico prediction of missense variants was performed by Polyphen-2 and SIFT (23, 24), splicing mutations by Spliceman (25). The variants were classified according to the latest American College of Medical Genetics guidelines (26).

### Statistical Analysis

Data was presented as mean ± SD or median (range) as appropriate. All statistical analyses were performed using Statistical Product and Service Solutions (SPSS) for window version 19.0 (SPSS Inc., Chicago, IL). Differences between two groups were compared with Student’s t test and the Mann-Whitney U test for normally distributed variables and non-normally distributed variables, respectively. Categorical data were presented as frequencies and percentages (%) and analyzed using the chi-square test and Fisher’s exact test. p<0.05 was considered as significant.

## Results

### *AQP2* Mutations in 7 Chinese NDI Families

**Figure 1:**
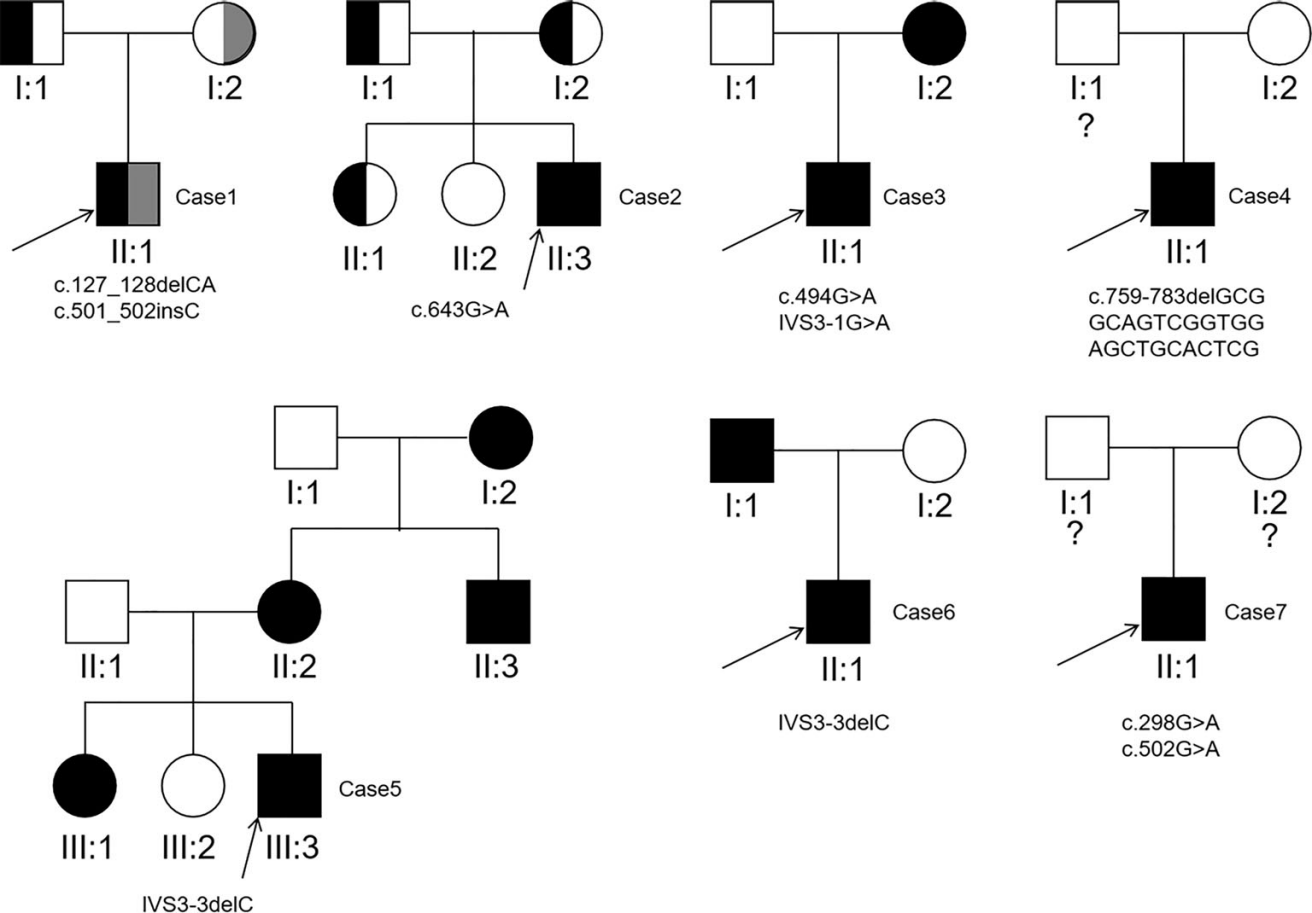
Pedigrees of the 7 families detected to carry pathogenic *AQP2* mutations. The question mark indicates *AQP2* mutation analysis of the individual was not performed.

**Figure 2:**
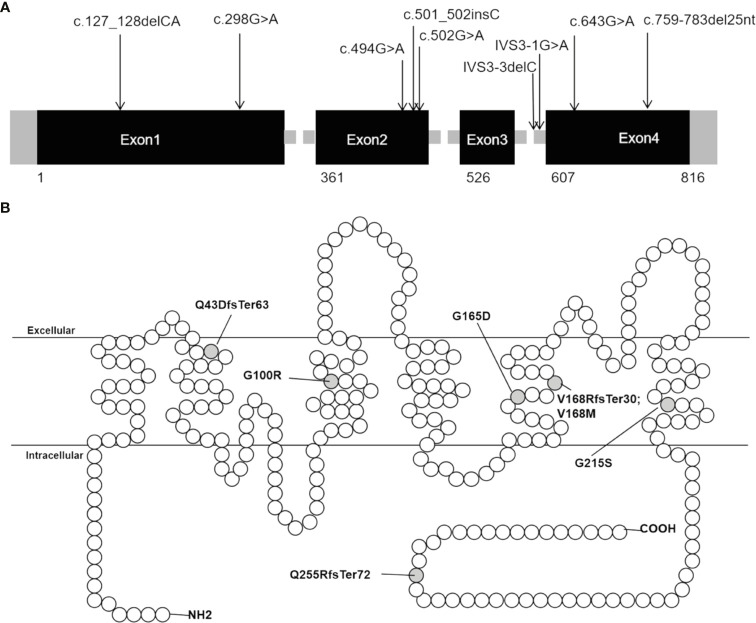
Distribution of *AQP2* mutations detected in our patients with NDI. **(A)** Distribution of the nine mutations in *AQP2* gene. **(B)** Schematic diagram of the primary structure of AQP2, showing the location of changed amino acid. The black rectangles indicate DNA coding sequence; the empty circles indicate amino acid of *AQP2*; the grey half-filled circles indicate amino acid alterations found in our study.

Sanger sequencing was applied to screen *AQP2* in 25 individuals (7 probands, 13 patients and their relatives). A total of nine different types of *AQP2* mutations were identified in 7 families (Details shown in **Table 1**). Missense mutations were the most common mutation type, followed by splicing mutations, frameshift mutations caused by small deletion or insertion. Of these *AQP2* mutations, 3 were novel mutations (p.G165D, p.Q255RfsTer72 and IVS3-3delC) (**Table 1**), which were predicted to be pathogenic according to ACMG guidelines. The pedigrees of these NDI families were shown in **Figure 1**. One novel splicing mutation caused by small deletion in intron3 (IVS3-3delC) was shared by 2 independent families (Family5 and Family6), and inherited in an autosomal dominant pattern. One novel frameshift mutation (p.Q255RfsTer72) involving C-terminal of AQP2 was also dominant. The other mutations were recessive and NDI patients carried compound heterozygous mutations (e.g., Case3: p.G165D and IVS3-1 G>A) or homozygous mutations (e.g., Case2: p.G215S). Most mutations were inherited from parents, while Case3 had a *de novo* mutation (IVS3-1G>A). The distribution of nine *AQP2* mutations was shown in **Figure 2**. Three out of the nine mutations were located in exon2, two in exon1, two in exon4, and two in intron3 (**Figure 2A**). The locations of amino acids were shown in the schematic diagram of AQP2, three in TMD5 (transmembrane domain-5), one in TMD-2, one in TMD-3, one in TMD-6 and one in ICT (intracellular C terminal) (**Figure 2B**).

**Table 1:** Mutation analysis of *AQP2* in patients with NDI.

No.	Type of mutation	Exon/Intron No.	Nucleotide change	Amino acid change	Amino acid location	Het/Hom	Inheritance	Family history	ACMG classification	Reported
1	Frameshift	Exon1; Exon2	c.127_128delCA; c.501_502 insC	p.Q43DfsTer63; p.V168RfsTer30	TMD-2;	Compound Het	Maternal; Paternal	No	Pathogenic;	Yes;
					TMD-5				Pathogenic	Yes
2	Missense	Exon4	c.643G>A	p.G215S	TMD-6	Hom	Maternal; Paternal	No	Pathogenic	Yes
3	Missense; Splicing	Exon2; Intron3	c.494G>A; IVS3-1G>A	p.G165D;	TMD-5; N.A.	Compound Het	Maternal; De novo	No	Pathogenic;	No;
				N.A.					Pathogenic	Yes
4	Frameshift	Exon4	c.759-783delGCGGCAGTCGGTGGAGCTGCACTCG	p.Q255RfsTer72	ICT	Het	N.A.	No	Pathogenic	No
5	Splicing	Intron3	IVS3-3delC	N.A	N.A	Het	Maternal	Yes	Pathogenic	No
6	Splicing	Intron3	IVS3-3delC	N.A	N.A	Het	Paternal	Yes	Pathogenic	No
7	Missense; Missense	Exon1; Exon2	c.298G>A; c.502G>A	p.G100R; p.V168M	TMD-3; TMD-5	Compound Het	N.A.	No	Pathogenic; Pathogenic	Yes;
										Yes

TMD, transmembrane domain; ICT, intracellular C terminal; N.A., Not available.

### Clinical Presentations in NDI Patients

Detailed demographic data, manifestations, biochemical characteristics and imaging results of the 7 probands with congenital NDI were reviewed and described. The demographic features and manifestations were shown in **Table 2**. All probands included were male, and 4 out of 13 patients were female in all families. The mean age of patients was 14.1 years (14.1 ± 13.8, range 2-42 years). The onset age was younger than 1 year old in all patients. The most common clinical presentations were polydipsia and polyuria (7/7) and intermittent fever (6/7). Among 7 patients, 3 had short stature and 1 had mental impairment.

**Table 2:** Clinical characteristics in patients with NDI.

No.	Sex	Onset-age (months)	Age (years)	Polydipsia and polyuria	Intermittent fever	Short stature	Mental impairment
1	Male	1	5	+	+	+	–
2	Male	4	8	+	+	+	–
3	Male	3	8	+	–	–	–
4	Male	3	12	+	+	–	+
5	Male	2	2	+	+	+	–
6	Male	6	22	+	+	–	–
7	Male	1	42	+	+	–	–

Y, years; M, months.

### Biochemical and Imaging Characteristics in NDI Patients

Biochemical and imaging characteristics were shown in **Table 3**. The urine output was increased in all patients, ranging from 4.3 ml/kg/h to 6.9ml/kg/h in children and adolescents, and 12.8 ml/kg/h to 13.9 ml/kg/h in adults. Urine SG and osmolality were decreased in all patients (6/6 and 7/7, respectively). However, serum osmolality and sodium were maintained at a relatively high level (307±23 mOsm/kgH_2_O; 145±6 mmol/L). Level of UA was elevated in 2 patients, and level of creatinine was normal in all patients (7/7). The urological ultrasonography was assessed in 6 patients except for the youngest child. Mild hydronephrosis of the left kidney and mild dilation of the right ureter were found in Case4. Hydronephrosis and dilation of the kidney and ureter as well as irregularly thickened bladder wall were found in Case6 and Case7.

**Table 3:** Biochemical and radiological characteristics in patients with NDI.

No.	Age, years	The Urine Output, ml/kg/h	Urine SG (1.005-1.030)	Urine osmolality, mOsm/kgH_2_O	Serum osmolality, mOsm/kgH_2_O	Serum Na, mmol/L (135-145)	Serum Cr(E), μmol/L(18-62)	Serum UA, μmol/L(210-416)	Urological ultrasonography
1	5	5.3	1.002↓	50	350	154↑	Normal	Normal	Normal
2a	8	11.3	1.002↓	157	307	143	Normal	Normal	Normal
3a	8	6.5	1.002↓	63	304	149↑	Normal	504↑	Normal
4a	12	6.9	N.A.	110	N.A.	142	Normal	459↑	Mild hydronephrosis of the left kidney; mild dilation of the right ureter
5	2	4.3	<1.005↓	146	285	137	Normal	Normal	N.A.
6	22	12.8	1.000↓	65	290	140	Normal	Normal	Hydronephrosis and dilation of the right kidney and ureter; irregularly thickened bladder wall
7	42	13.9	1.001↓	60	308	148↑	Normal	Normal	Hydronephrosis in the right kidney; dilation of the left ureter; irregularly thickened bladder wall

a means patients receiving drugs when evaluated.

### Drug Treatment

According to available data, for Case1, low-salt diet and good habits of drinking and voiding were recommended. Oral administration of hydrochlorothiazide (12.5 mg) and amiloride (1.25 mg) were given four times per day. The urine output and serum sodium were decreased when he visited us again after 3 months (5 to 3 L/24hours; 154 to 150 mmol/L). For Case6, hydrochlorothiazide (25 mg) and amiloride (2.5 mg) were given four times per day. The urine volume in 24h was decreased (20 to 9 L), urine osmolarity was increased (65 to 134 mOsm/kg H_2_O), and serum sodium was decreased (140 to 138 mmol/L) at the second visit.

### Genotype-Phenotype Correlation Analysis

Level of serum sodium in NDI patients with compound het mutations was higher than non-compound het mutations (150.3 ± 3.2 vs 140.5 ± 2.6 mmol/L, p=0.007, shown in **Supplementary Table 2**). There was no significant difference in onset-age, urine osmolality or other manifestations between two groups (**Supplementary Table 2**).

## Discussion

To the best of our knowledge, this is the first and largest case series of congenital NDI caused by *AQP2* mutation in Chinese population. So far, there were 71 putative disease-causing *AQP2* mutations reported to cause NDI (February 25^th^, 2021; The Human Gene Mutation Database at the Institute of Medical Genetics in Cardiff; http://www.hgmd.cf.ac.uk), including 55 missense/nonsense mutations, 4 splicing mutations, 9 small deletions, 2 small insertions, and 1 gross mutation. Here, we established genetic profile of *AQP2* in our patients, including 4 missense mutations, 2 splicing mutations and 3 frameshift mutations, showing a relatively even distribution in multiple domains. Further, we identified 3 novel *AQP2* mutations (p.G165D, p.Q255RfsTer72 and IVS3-3delC), which updated *AQP2* mutation spectrum and provided basis for functional studies and drug development.

Among these mutations, seven out of nine *AQP2* mutations caused recessive NDI and two caused dominant NDI. The novel missense mutation (G165D, Case3) exhibiting a recessive mode was located in transmembrane domain-5 of AQP2 and predicted to be pathogenic by software, which may lead to retention in the endoplasmic reticulum (ER), the main molecular pathogenesis by recessive mutations reported previously (13). The novel frameshift mutation (p.Q255RfsTer72, Case4) located in the C-terminal and the novel splicing mutation (IVS-3delC) identified in two independent families with NDI (Case6 and Case7) exhibited dominant pattern, which may cause misrouting of AQP2 heterotetramer formed by wild-type and mutant monomer according to previous studies (17, 20). The heterozygous (p.Q43DfsTer63 and p.V168RfsTer30, Case1) and homozygous mutations (G215S, Case2) were reported by us before (27). The splicing mutation (IVS3-1G>A) was identified in a sporadic patient by other groups (28). Missense mutations (G100R and V168M) were predicted to impair monomer folding or pore features by previous studies (18, 29, 30). All mutations identified in our patients explained the NDI phenotype and provided persuasive evidence for studying molecular biology of AQP2. There were also limitations in this study, including the lack of next-generation sequencing and molecular characterization of mutations. Clinical characteristics of NDI patients were described and genotype-phenotype correlation analysis was performed. We firstly found level of serum sodium was higher in patients with compound het *AQP2* mutations than non-compound het mutations, suggesting more severe phenotype due to more damage to AQP2 tetramer functions, which may be explained by functional analysis in a larger sample population (20). In our patients, the onset-age was younger than 1 year old. Common presentations were polydipsia, polyuria, and intermittent fever, and biochemical features were characterized by high osmotic hypernatremia and low osmotic urine. Results also indicated elevated level of serum uric acid was found in 2 cases, possibly due to inhibition of uric acid excretion by hydrochlorothiazide. Dilation of the urinary tract was found in 3 of 7 patients and the youngest was only 12 years old, which was a severe complicaton of NDI, especially common in patients with poor voiding habits. Anatomic obstructions should be considered in patients with hydronephrosis (1, 31). Compared to previous studies (32–35), we characterized phenotypes from more cases in a different population and found similar spectrum.

For treatment in NDI, low-salt diet, good habits of drinking and voiding were recommended. Adequate hydration should be noted for infants. Hypotonic fluids are usually appropriate for intravenous infusion, but hyponatremia should be avoided when other salt losses exist, such as diarrhea. Common drugs included hydrochlorothiazide and amiloride (7, 17, 36). Thiazides blocked sodium-chloride cotransporter in the distal convoluted tubule and activated renin-angiotensin-aldosterone system, leading to increased sodium and water resorption in proximal tubule and decreased urine excretion (37). Amiloride was a combination drug with thiazides to prevent hypokalemia. Here, urine output was decreased by nearly 50% after treatment with hydrochlorothiazide in two cases, and level of serum sodium was decreased. However, lack of long-term follow-up data and the limited sample size were also limitations. Novel treatments including molecular chaperones and gene therapy may cure congenital NDI, but few clinical data are currently available (7, 17, 36).

In conclusion, we performed gene mutation analysis in 7 Chinese families and identified 9 *AQP2* mutations, including 3 novel mutations (p.G165D, p.Q255RfsTer72 and IVS3-3delC). Then, we characterized clinical, biochemical and imaging features in these patients, and found similar phenotype spectrum compared to previous study. Further, we found level of serum sodium was higher in patients with compound het *AQP2* mutations than non-compound het mutations. This knowledge broadens genotypic and phenotypic spectrum for rare congenital NDI and provided basis for studying molecular biology of AQP2.

## Data Availability Statement

The raw data supporting the conclusions of this article will be made available by the authors, without undue reservation.

## Ethics Statement

The studies involving human participants were reviewed and approved by the Institutional Review Board and Ethics Committee of PUMCH. Written informed consent to participate in this study was provided by the participants’ legal guardian/next of kin.

## Author Contributions

Study design: LD and WX. Study conduct: QL, DT, and JC. Data collection: QL, DT, and JC. Data analysis: QL, DT, JC, LD, and WX. Data interpretation: QL, DT, JC, LD, and WX. Drafting manuscript: QL. Revising manuscript content: QL, DT, JC, LD, and WX. Approving final version of manuscript: QL, LD, and WX. All authors contributed to the article and approved the submitted version.

## Funding

This work was supported by National Natural Science Fund (No. 81670814), National Key R&D Program of China (2018YFA 0800801) and National Natural Science Fund (No. 81970757).

## Conflict of Interest

The authors declare that the research was conducted in the absence of any commercial or financial relationships that could be construed as a potential conflict of interest.

## Supplementary Material

Supplementary Table 1 The PCR primer sets and conditions for *AQP2 and AVPR2* gene.

Supplementary Table 2 Comparison of phenotypes in patients with compound het *AQP2* mutations and non-compound het mutations. Data presents as Mean ± SD or Median (minimum, maximum), p<0.05 was considered as significant.
