# Supplementary material for: Novel AQP2 Mutations and Clinical Characteristics in Seven Chinese Families With Congenital Nephrogenic Diabetes Insipidus
Source: Front Endocrinol (Lausanne). 2021 Jun 10;12:686818. doi: 10.3389/fendo.2021.686818 (PMC8225504; doi:10.3389/fendo.2021.686818)
Supplement: Supplementary Table 1 — The PCR primer sets and conditions for AQP2 and AVPR2 gene. [file DataSheet_1.docx]

| **Gene** | **Exon** | **Primer sequence (5’ to 3’)** | **AT (℃)** | **Size (bp)** |
| --- | --- | --- | --- | --- |
| *AQP2* | Exon 1 | F: AGGTATTGGCCTCAACGACTC | 61 | 797 |
|  |  | R: TGAGGCTCTTCCTGCACGAC |  |  |
|  | Exon 2+3 | F: CCTCAGGCCCCAATCTAAT | 61 | 947 |
|  |  | R:AGGAGGAGTGTGGAGGGTTT |  |  |
|  | Exon 4 | F: GCTGGCGTTGTCGTTGTA | 62 | 793 |
|  |  | R: GAGAACTTGGGGATGAACACA |  |  |
| *AVPR2* | Exon 1 | F: ATCCTGGGTTCTGTGCATCCGT | 60 | 388 |
|  |  | R: CTCCCCTCCCCACTCATTG |  |  |
|  | Exon 2A | F: CATGAGCCTGGGGTGTGTATCC | 63 | 696 |
|  |  | R: CGCTGCCACCTTCCACGTTG |  |  |
|  | Exon 2B | F: TGCCTCCTCCTACATGATCCTG | 62 | 677 |
|  |  | R: GGCCAGCAACATGAGTAGCAC |  |  |
|  | Exon 3 | F: GCCAAGACTGTGAGGATGACG | 62 | 688 |
|  |  | R: TGCAGCCCCTCCTACACC |  |  |

**Supplementary Table 1. The PCR primer sets and conditions for AQP2 and AVPR2 gene.**
