# Supplementary material for: Novel AQP2 Mutations and Clinical Characteristics in Seven Chinese Families With Congenital Nephrogenic Diabetes Insipidus
Source: Front Endocrinol (Lausanne). 2021 Jun 10;12:686818. doi: 10.3389/fendo.2021.686818 (PMC8225504; doi:10.3389/fendo.2021.686818)
Supplement: Supplementary Table 2 — Comparison of phenotypes in patients with compound het AQP2 mutations and non-compound het mutations. Data presents as Mean ± SD or Median (minimum, maximum), p<0.05 was considered as significant. [file DataSheet_2.docx]

|  | **Compound het mutation group** | **Non-compound het mutation group** | **p value** |
| --- | --- | --- | --- |
| Onset age, months | 1.0 (1.0, 3.0) | 3.5 (2, 6) | 0.114 |
| Age, years | 18.5±20.4 | 11±8.4 | 0.527 |
| The urine output, ml/kg/h | 8.6±4.7 | 8.8±3.9 | 0.939 |
| Serum Na,  mmol/L | **150.3±3.2** | **140.5±2.6** | **0.007** |
| Serum osmolality, mOsm/kgH_2_O | 320.7±25.5 | 294.0±11.5 | 0.200 |
| Urine osmolality, mOsm/kgH_2_O | 57.8±6.8 | 119.5±41.5 | 0.055 |
| Short stature | 33% (1/3) | 50% (2/4) | 1.000 |
| Mental impairment | 0% (0/3) | 25% (1/4) | 1.000 |
| Uric acid abnormality | 33% (1/3) | 50% (2/4) | 1.000 |
| Urological abnormality | 67% (2/3) | 67% (2/3) | 1.000 |

**Supplementary Table 2. Comparison of phenotypes in patients with compound het AQP2 mutations and non-compound het mutations.**

Data presents as Mean±SD or Median (minimum, maximum), p<0.05 was considered as significant.
